# The Clinical Significance of the CD163+ and CD68+ Macrophages in Patients with Hepatocellular Carcinoma

**DOI:** 10.1371/journal.pone.0059771

**Published:** 2013-03-29

**Authors:** Ling-Qun Kong, Xiao-Dong Zhu, Hua-Xiang Xu, Ju-Bo Zhang, Lu Lu, Wen-Quan Wang, Qiang-Bo Zhang, Wei-Zhong Wu, Lu Wang, Jia Fan, Zhao-You Tang, Hui-Chuan Sun

**Affiliations:** 1 Liver Cancer Institute and Zhongshan Hospital, Fudan University, the Key Laboratory for Carcinogenesis & Cancer Invasion, The Ministry of Education of China, Shanghai, P.R. China; 2 Department of Pancreatic and Hepatobiliary Surgery, Fudan University Shanghai Cancer Center, Shanghai, P.R. China; University of Modena & Reggio Emilia, Italy

## Abstract

Our previous study has found that the abundance of peritumoral CD68^+^ macrophages was associated with poor prognosis in hepatocellular carcinoma (HCC) after resection. However, CD68 staining could not discriminate the protumoral or tumoricidal subpopulations from pan-macrophages. CD163 is a marker of alternatively activated macrophages. In this study, the clinical significance of CD163^+^ cells in tumors and peritumoral liver tissues was evaluated in a cohort of 295 patients with HCC after curative resection. We found that the density of CD163^+^ cells was well correlated with that of CD68^+^ cells in both tumors and peritumoral liver tissues but was much more. Immunostaining on consecutive sections and flow cytometry assay on surgical resected specimens further supported the findings that the CD163^+^ cells was more abundant than CD68^+^ cells. The density of peritumoral CD68^+^ cells was associated with poor recurrence-free survival (RFS) and poor overall survival (OS) (*P* = 0.004 and *P* = 0.001, respectively), whereas the CD163^+^ cells have no prognostic values either in tumors or in peritumoral liver tissues. In another cohort of 107 HCC patients, preoperative plasma concentration of soluble form of CD163 (sCD163) was associated with active hepatitis-related factors but not associated with the markers of tumor invasion. In conclusion, both the CD163^+^ cells local infiltration and plasma sCD163 were of limited significance in HCC, and they were more likely markers related to active hepatitis rather than tumor progression.

## Background

Hepatocellular carcinoma (HCC), accounting for 70% to 85% of primary liver cancer, is one of the most prevalent cancers worldwide. It ranks as the second leading cause of cancer deaths for men and the sixth for women and half of these cases and deaths were estimated to occur in China. [1] Although hepatectomy is one of the best methods to provide long-term survival for patients with HCC, [2] a high recurrence rate after surgery is a major problem. Tumor microenvironment, especially macrophages, which play an important role in the initiation, progression, and metastasis of various tumors, has been intensively studied in recent years [3], [4].

Macrophages constitute a major component of the infiltrates in most solid tumors. In several solid tumors, for example, breast, prostate, endometrium, bladder, kidney, esophagus and lymphomas, the intratumoral infiltrated macrophages were associated with poor prognosis. [5]–[11] Our previous study also found that the abundance of CD68^+^ macrophages infiltrated in peritumoral liver tissue, but not in tumor, was associated with poor prognosis for the patients with HCC after curative liver resection. [12] Macrophages could be divided into M1 (or classically activated) and M2 (or alternatively activated) subpopulations according to their functional phenotypes [13]. It is now generally accepted that tumor-associated macrophages have an M2 phenotype with pro-tumoral effects. [14] However, CD68, the most widely used marker for macrophages, could not distinguish M1 or M2 subtypes from all the infiltrated macrophages (pan-macrophages). [15].

CD163, a member of the scavenger receptor cysteine-rich family, is a 130-kDa transmembrane protein. [16], [17] CD163 was reported to be expressed almost exclusively on circulating monocytes and macrophages. [18]–[20] CD163 was involved in anti-inflammatory functions and predominantly expressed on M2 macrophages. [21]–[24] Intratumoral CD163^+^ macrophages counts were reported to be associated with poor prognosis in various tumors, such as melanoma (in tumor stroma), [25] nongynecologic leiomyosarcoma, [26] and renal cell carcinoma [27]. Based on these reports, we hypothesized that CD163^+^ macrophages were a subpopulation of CD68^+^ pan-macrophages and may be more suitable to identify the tumor-promoting macrophages. Using the marker CD163, we could further discriminate the patients with different prognosis. In this study, the distribution and clinical significance of macrophages in tumor and peritumoral liver tissues were evaluated with the markers of CD163 and CD68, and the differences between these two markers were analyzed.

Furthermore, the soluble form of CD163 (sCD163) could be released from active macrophages and was found in serum when macrophages were activated. Overexpression of sCD163 could be a marker of a progressive inflammatory milieu. [28]–[30] Serum sCD163 was significantly elevated in patients with fulminant hepatic failure as compared with patients with hepatitis or healthy controls [31]; it was also reported to be a predictor of mortality for patients with acute liver failure [32]. Patients with HCC often complicated with hepatitis and/or liver cirrhosis. Therefore, the clinical relevance of CD163 expression in liver tissue and the sCD163 level in peripheral blood is of particular interest. In the current study, plasma sCD163 levels was also evaluated in another cohort of patients with HCC and their associations with the clinical features of tumor progression and active hepatitis were explored.

## Patients and Methods

### Patients, Specimens, and Follow-up

Surgical specimens from 295 consecutive patients who underwent curative liver resection in our institute (Liver Cancer Institute, Fudan University) with pathologically confirmed HCC were collected as cohort 1. Plasma samples from another 107 patients with HCC (cohort 2) were also collected before surgery and stored at –80°C until use. Tumor stage was determined according to the 2009 International Union Against Cancer TNM classification system (7th edition). [33] Tumor differentiation was graded by the Edmondson grading system. Hepatitis B history was defined as ever detectable serum hepatitis B surface antigen. Microvascular invasion was defined as the presence of tumor cells within a vascular lumen lined by endothelium under microscopy. [34].

Follow-up procedures for the patients after surgery were described in our previous reports. [35], [36] Briefly, patients were monitored by abdominal ultrasonography, serum α-fetoprotein (AFP), and chest radiography with an interval of 2 to 6 months according to the postoperative time. If recurrence was suspected, computed tomography scanning or magnetic resonance imaging was performed immediately. Treatment modalities after relapse were administered according to a uniform guideline. [36]–[38] Overall survival (OS) or recurrence-free survival (RFS) was defined as the interval between surgery and death or disease recurrence, respectively. If recurrence was not diagnosed, patients were censored on the date of death or the last follow-up. This study was approved by the Zhongshan Hospital Research Ethics Committee. Informed consent was obtained from each patient according to the committee’s regulation. Participants provided their written informed consent to participate in this study.

### Evaluation of Immunohistochemistry and Enzyme-linked Immunosorbent Assay

Two cores were taken from representative formalin-fixed paraffin-embedded tumor tissue and liver tissue from the patients of cohort 1 to construct tissue microarray slides (in collaboration with Shanghai Biochip Company, Shanghai, China). Tumor samples were taken from the nonnecrotic peripheral zone of the tumor. Liver samples were taken from the peritumoral liver tissue within a distance of 1 cm away from the tumor margin. Duplicate cylinders from 2 different areas, a total of 4 punches from each patient, were obtained. Sections were constructed with tumors and matched peritumoral samples. The immunohistochemistry protocols and the evaluation of the immunostaining were described in our previous studies. [35] Primary antibodies were mouse anti-human antibodies combined with CD163 (1∶100 dilution; AbD Serotec, Oxford, UK) and CD68 (1∶100 dilution; Abcam, Cambridge, MA). Under high-power magnification (×200), photographs of 2 representative fields of each punch were captured by a computerized image system composed of a Leica CCD camera DFC 500 connected to a Leica DM IRE2 microscope and Leica Qwin Plus v3 software (Leica Microsystems Imaging Solution, Cambridge, UK). Identical settings were used for all photographs. The area of positive staining was measured in pixels by Image-Pro Plus v6.2 software (Media Cybernetics, Bethesda, MD) as described. [12] The densities of CD163 and CD68 staining were expressed as the ratio of positive staining area to the total area of each photograph and the cell densities were taken as surrogate measures for the counts of CD163^+^ and CD68^+^ cells.

Plasma sCD163 levels of the patients from cohort 2 were measured by an enzyme-linked immunosorbent assay kit (R&D Systems, Minneapolis, MN) according to the user’s manual.

### Cell Suspension Solution Preparation and Flow Cytometric (FCM)Analysis of CD68 and CD163

Single cell suspension were obtained from three paired HCC tissue and surrounding non-tumoral liver tissue from surgical resection samples. Specimens were minced with scissors and digested by incubation for 1 h at 37°C in high-glucose DMEM containing 0.1% collagenase IV (Sigma-Aldrich, St. Louis, MO). After being washed in medium plus 10% fetal bovine serum, the cell suspension was forced through a graded series of meshes to separate the cell components from stroma and aggregates. After that, cells were fixed by 4% formaldehyde for 10 min, and then bursting of cell membranes by using 0.05% of the tritonX-100 for 15 min, after that, cells were incubated for 30 min at 4°C with CD163and CD68 or with a control in PBS containing 0.5% (w/v) BSA. Cells were analyzed on a fluorescent activated cell sorter (BD Biosciences, San Jose, CA). We used primary murine monoclonal antibodies against human CD68 conjugated to FITC, and human CD163conjugated to allophycocyanin (APC). FITC-CD68 and APC-CD163 antibodies were purchased from Biolegend (San Diego, CA).

### Statistical Analysis

Analysis was performed with SPSS13.0 for Windows (SPSS). The Pearson χ^2^ test or Fisher exact test was used to compare qualitative variables, and quantitative variables were analyzed by the Student *t* test or Spearman correlation test. Kaplan-Meier analysis was used to determine the survival. The log-rank test was used to compare the survival between subgroups, and the Cox regression model was used to perform multivariate analysis. *P*<0.05 was considered statically significant.

## Results

### Demographics

Table 1 lists the demographic data of patients from cohorts 1 and 2. At the time of the last follow-up of cohort 1, 138 patients had tumor recurrence and 123 patients had died, including 35 patients who died of liver failure without tumor recurrence. The 1-, 3- and 5-year OS rates were 88%, 64%, and 58%, respectively, and the 1-, 3- and 5-year recurrence rates were 25%, 43%, and 47%, respectively. Because the median follow-up time for patients in cohort 2 was less than 12 months, follow-up data were not analyzed for this cohort.

**Table 1:** Clinicopathologic features of patients from cohort 1 and cohort 2.

Features	Values/counts	
	Cohort 1 (*n* = 295)	Cohort 2 (*n = *107)
Age, median (range), y	52 (22–80)	54 (28–77)
Gender, male/female	247/48	91/16
α-Fetoprotein, median (range), ng/dL	185 (0–60,500)	61.75 (1–60,500)
Liver cirrhosis, yes/no	227/68	90/17
Hepatitis B history, yes/no	230/65	91/16
Hepatitis B e antigen, positive/negative	111/184	31/76
Tumor size, mean ± SD, cm	5.58±3.93	5.41±3.43
Encapsulation, complete/none	146/149	52/55
Tumor differentiation, I–II/III–IV	202/93	75/31
Microvascular invasion, yes/no	119/176	47/60
TNM stage, I/II/IIIA	157/118/20	72/8/27

Abbreviations: y, year.

### Immunohistochemical Findings in Tissue Microarray

**Figure 1:**
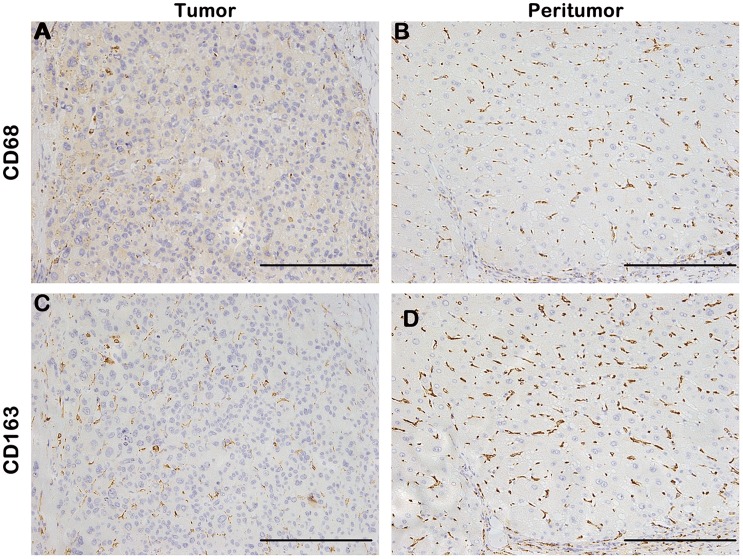
Representative immunostaining of CD163 and CD68 on consecutive sections of paired tumor tissue and peritumoral liver tissue. The densities of intratumoral CD68^+^ cells (A) and CD163^+^ cells (C) were lower in tumor than in corresponding peritumoral liver tissue (B and D). The density of CD163^+^ cells was higher than that of CD68^+^ cells in both tumor and in peritumoral liver tissue. Scale bar, 200 µm.

**Figure 2:**
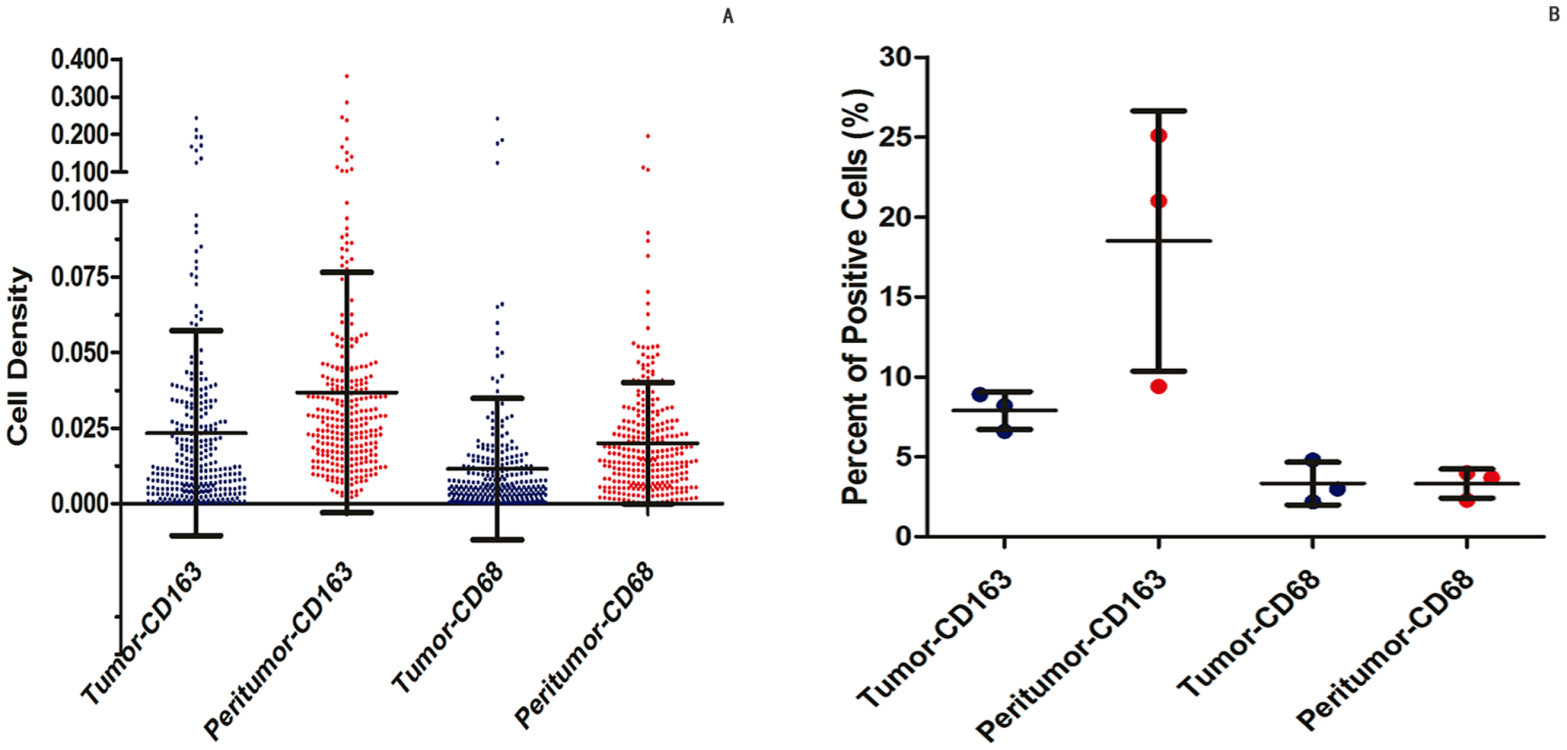
The dot plots of the densities of CD163^+^ and CD68^+^ cells in tumor and in peritumoral liver tissue from the 295 patients (cohort 1). (A) The dot plots of ratios of CD163 and CD68-expressing cells to total cells in 3 paired tumors and peritumoral liver tissues which were counted by flow cytometry (B). Error bars, S.D.

CD68 and CD163 staining appeared mainly in the cytoplasm of stroma cells in tumor and peritumoral liver tissue. Most tumor cells and hepatic cells were negatively stained, although sporadic positive staining on these cells could also be observed (Figure 1). The average densities of CD163 and CD68 staining were 0.023±0.034 and 0.011±0.023 in tumor, respectively, and 0.037±0.040 and 0.020±0.020 in peritumoral liver tissue, respectively (Figure 2A). The densities of CD68^+^ and CD163^+^ cells in peritumoral liver tissue were significantly higher than those within tumor (*P*<0.001 for both).

### The Clinical Relevance of the CD163^+^ and CD68^+^ Macrophages

The density of intratumoral CD163^+^ macrophages was positively correlated with patient’s age and serum alkaline phosphatase (ALP) concentration, whereas the density of intratumoral CD68^+^ macrophages was only correlated with the patient’s age. Both the peritumoral CD163^+^ and CD68^+^ macrophages were associated with the hepatitis-related features, such as serum aspartate aminotransferase (AST) and γ-glutamyl transpeptidase (γ-GT), and the tumor-related features, including serum α-fetoprotein (AFP), tumor size, and presence of microvascular invasion; the density of peritumoral CD68^+^ macrophages was also associated high TNM stage (Tables 2 and 3).

**Table 2:** Relationships between intratumoral/peritumoral CD163 expression and clinicopathologic features.

Variables	Intratumoral CD163 expression		Peritumoral CD163 expression	
	Average level or correlationcoefficient*	*P*	Average level or correlationcoefficient*	*P*
Age, y	0.184	0.001	0.001	0.984
Gender, male vs female	0.025±0.036 vs. 0.017±0.024	0.155	0.037±0.040 vs. 0.033±0.040	0.499
Hepatitis B history, yes vs. no	0.023±0.032 vs. 0.025±0.040	0.665	0.037±0.043 vs. 0.037±0.027	0.963
Hepatitis B e antigen, positive vs.negative	0.023±0.035 vs. 0.023±0.034	0.933	0.040±0.036 vs.0.035±0.042	0.325
ALT	−0.019	0.744	0.051	0.387
AST	0.047	0.429	0.136	0.021
γ-GT	0.048	0.409	0.139	0.017
ALP	0.125	0.033	0.103	0.079
Liver cirrhosis, yes vs. no	0.023±0.037 vs. 0.023±0.023	0.930	0.039±0.042 vs. 0.030±0.031	0.087
α-Fetoprotein	0.001	0.987	0.159	0.006
Tumor size	0.048	0.410	0.276	<0.001
Tumor encapsulation, complete vs. no	0.024±0.033 vs. 0.022±0.035	0.692	0.033±0.032 vs. 0.040±0.046	0.165
Microvascular invasion, yes vs. no	0.022±0.028 vs. 0.024±0.037	0.615	0.043±0.047 vs. 0.033±0.034	0.037
Tumor differentiation, I–II vs. III–IV	0.023±0.034 vs. 0.023±0.033	0.973	0.036±0.039 vs. 0.038±0.042	0.642
TNM stage, I vs. II/IIIA	0.024±0.039 vs. 0.022±0.027	0.516	0.033±0.035 vs. 0.041±0.044	0.085

* If the clinicopathologic variable is a quantitative one, Spearman correlation analysis was performed and the correlation coefficient was presented.

Abbreviations: y, year; ALT, alanine aminotransferase; AST, aspartate aminotransferase; γ-GT, γ-glutamyl transpeptidase; ALP, alkaline phosphatase.

**Table 3:** Relationships between intratumoral/peritumoral CD68 expression and clinicopathologic features.

Variables	Intratumoral CD68 expression		Peritumoral CD68 expression	
	Average level or correlation coefficient*	*P*	Average level or correlation coefficient*	*P*
Age	0.148	0.011	−0.029	0.623
Gender, male vs. female	0.012±0.025 vs. 0.008±0.010	0.297	0.020±0.018 vs. 0.021±0.028	0.733
Hepatitis B history, yes vs. no	0.012±0.024 vs. 0.010±0.023	0.590	0.020±0.021 vs. 0.021±0.017	0.503
Hepatitis B e antigen, positive vs. negative	0.011±0.026 vs. 0.012±0.022	0.822	0.021±0.024 vs. 0.019±0.018	0.449
ALT	0.013	0.825	−0.013	0.823
AST	0.091	0.120	0.118	0.045
γ-GT	0.036	0.542	0.126	0.031
ALP	0.079	0.178	0.092	0.116
Liver cirrhosis, yes vs. no	0.012±0.026 vs. 0.008±0.010	0.225	0.021±0. 018 vs. 0.017±.0.025	0.123
α-Fetoprotein	0.062	0.285	0.183	0.002
Tumor size	0.039	0.505	0.389	<0.001
Tumor encapsulation,complete vs. no	0.012±0.025 vs. 0.011±0.022	0.683	0.018±0.016 vs. 0.022±0.023	0.093
Microvascular invasion, yes vs. no	0.014±0.029 vs. 0.010±0.019	0.192	0.024±0.025 vs. 0.017±0.015	0.005
Tumor differentiation, I–II vs.III–IV	0.009±0.014 vs. 0.016±0.037	0.103	0.020±0.018 vs. 0.020±0.025	0.745
TNM stage, I vs. II/IIIA	0.010±0.020 vs. 0.013±0.027	0.309	0.017±0.015 vs. 0.024±0.024	0.004

* If the clinicopathologic variable is a quantitative one, Spearman’s correlation analysis was performed and the correlation coefficient was presented.

Abbreviations: y, year; ALT, alanine aminotransferase; AST, aspartate aminotransferase; γ-GT, γ-glutamyl transpeptidase; ALP, alkaline phosphatase.

### CD163^+^ Cells were More Abundant than CD68^+^ Cells

In cohort 1, the density of CD163^+^ cells was positively correlated with that of CD68^+^ cells in tumor tissue (*r* = 0.417, *P*<0.001) and peritumoral liver tissue (*r* = 0.565, *P*<0.001). However, the average density of CD163^+^ cells was 22.83-fold and 4.11-fold higher in tumor and peritumoral liver tissue, respectively, compared with that of CD68^+^ cells. Immunostaining on consecutive sections of tumor tissue and peritumoral liver tissue also showed that the density of CD163^+^ cells was much higher than that of the CD68^+^ cells both in tumor and in peritumoral liver tissue (Figure 1).

FCM analysis also found that the ratio of CD163-expressing cells to total cells were higher than CD68 from surgical HCC specimens and non-tumoral surrounding liver tissue (*P* = 0.011 and *P* = 0.033, respectively, Figure 2B).

### Prognostic Significance of CD163 and CD68 Staining

**Figure 3:**
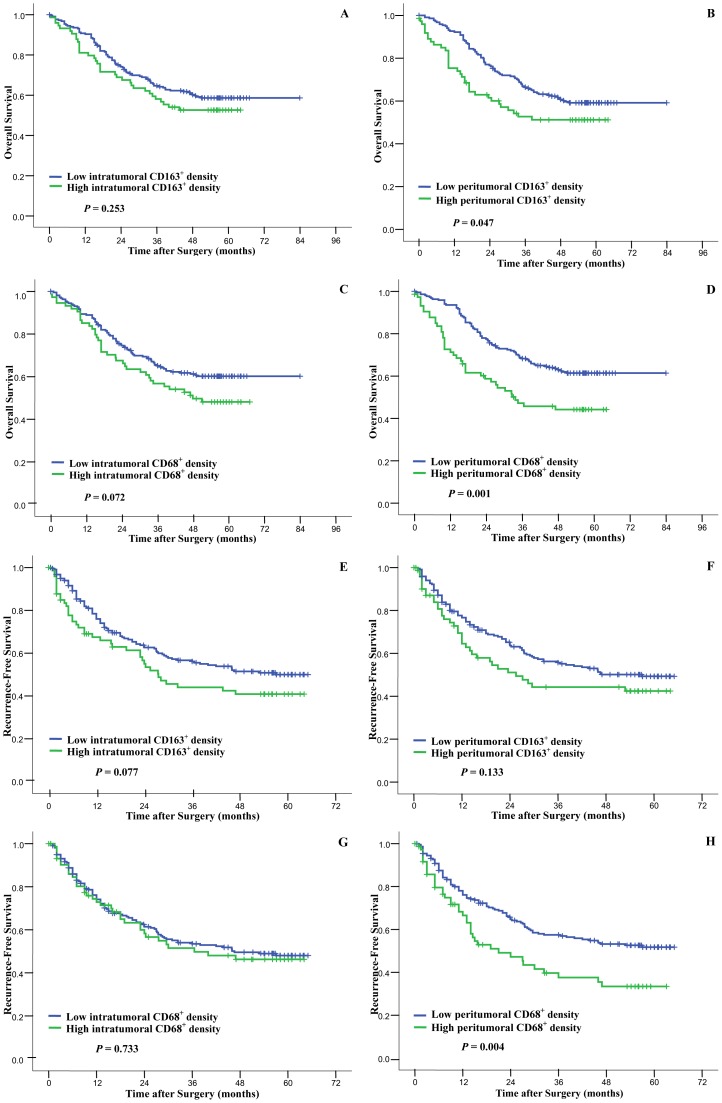
Cumulative overall survival (OS) and recurrence-free survival (RFS) curves of patients with high and low densities of CD163^+^ (panels A, B, E, F) or CD68^+^ (panels C, D, G, H) cells infiltrated in tumor and peritumoral liver tissue. Intratumoral densities of CD68^+^ cells and CD163^+^ cells could not discriminate patient with different OS and RFS (panels A, C, E, G). In peritumoral liver tissue, the densities of CD163^+^ and CD68^+^ cells were both associated with poor OS, and the density of CD68^+^ cells was also associated with RFS (panels B, D, F, H).

As shown in Table 4, the prognostic value of each clinicopathologic feature was analyzed in univariate and multivariate analysis. In the univariate analysis, serum AFP>200 ng/dL, serum γ-GT >50 U/L, tumor size >5 cm, presence of microvascular invasion, and advanced TNM stage were risk factors for both OS and RFS. Poor tumor differentiation, AST >75 U/L and ALP>140 U/L were also associated with poor OS. Using the 75th percentile value of the densities of CD163^+^ cells or CD68^+^ cells in cohort 1, we divided the patients into subgroups with a high or low macrophage density. Intratumoral CD163 expression was not associated with OS (*P* = 0.253, Figure 3A) or RFS (*P* = 0.077, Figure 3E). CD163^+^ macrophage infiltration in peritumoral liver tissue was associated with poor OS (*P* = 0.047; median OS for patients with high and low CD163^+^ macrophages infiltration were 56.4 months and 64.7 months, respectively) but not RFS (*P* = 0.133, Figure 3B and 3F). In consistence with our previous study, [12] patients with high CD68^+^ cells infiltration in peritumoral liver tissue had a poor prognosis for both OS and RFS (*P* = 0.001 and *P* = 0.004, respectively), and the intratumoral CD68^+^ cell density had no prognostic significance (Figure 3 and Table 4).

**Table 4:** Univariate and multivariate analyses of factors associated with survival and recurrence.

Features	Overall survival			Recurrence-free survival		
	*P* *	Multivariate		*P* *	Multivariate	
		HR (95% CI)	*P*		HR (95% CI)	*P*
Age, ≤52 vs. >52 y	0.932		NA	0.888		NA
Gender, female vs. male	0.265		NA	0.193		NA
Hepatitis B history, yes vs. no	0.395		NA	0.827		NA
Liver cirrhosis, yes vs. no	0.169		NA	0.163		NA
α-Fetoprotein, >200 vs. ≤200 ng/dL	0.033	1.078 (0.733–1.586)	0.703	0.046	1.073 (0.752–1.531)	0.696
ALT, >75 vs. ≤75 U/L	0.408		NA	0.380		NA
AST, >75 vs. ≤75 U/L	<0.001	1.860 (1.105–3.130)	0.019	0.186		NA
γ-GT, >50 vs. ≤50 U/L	<0.001	1.636 (1.090–2.456)	0.018	0.001	1.396 (0.973–2.004)	0.070
ALP, >140 vs. ≤140 U/L	<0.001	1.635 (0.994–2.690)	0.053	0.911		NA
Tumor size, >5 vs. ≤5 cm	<0.001	1.906 (1.270–2.861)	0.002	<0.001	1.523 (1.052–2.256)	0.026
Tumor differentiation, III–IV vs. I–II	0.008	1.552 (1.075–2.241)	0.019	0.072		NA
Tumor encapsulation, complete vs. none	0.624		NA	0.448		NA
Microvascular invasion, yes vs. no	<0.001	1.045 (0.559–1.954)	0.889	<0.001	1.022 (0.560–1.867)	0.943
TNM stage, I vs. II vs IIIA	<0.001	1.918 (1.437–2.561)	<0.001	<0.001	1.858 (1.411–2.4446)	<0.001
Peritumoral CD68, high vs. low	0.001	1.518 (1.024–2.251)	0.038	0.004	1.602 (1.087–2.362)	0.017
Intratumoral CD68, high vs. low	0.072		NA	0.733		NA
Peritumoral CD163, high vs. low	0.047	0.800 (0.512–1.249)	0.326	0.133		NA
Intratumoral CD163, high vs. low	0.253		NA	0.077		NA

* univariate analysis.

Abbreviations: y, year; ALT, alanine aminotransferase; AST, aspartate aminotransferase; γ-GT, γ-glutamyl transpeptidase; ALP, alkaline phosphatase; NA, not adopted.

The above factors with a *P*<0.05 in the univariate analysis were further analyzed in multivariate Cox proportional hazard analysis. Peritumoral CD68^+^ cell density was an independent risk factor for OS and RFS (*P* = 0.038 and *P* = 0.017, respectively); whereas the CD163^+^ cell density was not (Table 4).

### Plasma sCD163 was a Marker of Hepatitis More than a Marker of Tumor Progression

The average level of preoperative plasma sCD163 was 74.43±30.01 ng/mL in the patients from cohort 2. High level of plasma sCD163 (using the 75^th^ percentile level as the cutoff value) was significantly associated with hepatitis-related factors, including serum alanine aminotransferase (ALT), AST, γ-GT, and ALP, but not tumor-related factors, such as tumor size, microvessel invasion, and TNM stage (Table 5).

**Table 5:** Relationships between plasma sCD163 and clinicopathologic features.

Variables	Average level or correlation coefficient*	*P*
Age, y	0.229	0.018
Gender, male/female	73.49±30.87 vs. 79.78±24.74	0.442
Hepatitis B history, yes/no	79.10±16.57 vs. 73.61±31.79	0.502
Hepatitis B e antigen, positive/negative	73.99±28.43 vs. 74.61±30.82	0.923
ALT	0.327	0.001
AST	0.491	<0.001
γ-GT	0.421	<0.001
ALP	0.421	<0.001
Liver cirrhosis, yes/no	76.79±31.21vs. 61.97±18.79	0.062
α-Fetoprotein	0.181	0.062
Tumor size	0.192	0.048
Tumor encapsulation, complete/no	71.91±31.04 vs. 76.82±29.09	0.401
Microvascular invasion, yes/no	78.95±31.24 vs. 70.89±28.78	0.174
Tumor differentiation, I–II/III–IV	71.74±30.63 vs. 79.84±27.94	0.192
TNM stage, I/II–IIIA	71.62±29.34 vs. 80.47±30.99	0.167

* If the clinicopathologic variable is a quantitative one, Spearman’s correlation analysis was performed and the correlation coefficient was presented.

Abbreviations: y, year; ALT, alanine aminotransferase; AST, aspartate aminotransferase; γ-GT, γ-glutamyl transpeptidase; ALP, alkaline phosphatase.

## Discussion

The results from our previous studies and others have indicated that the abundance of peritumoral infiltrated CD68^+^ macrophages was associated with a poor prognosis for patients with HCC who underwent curative liver resection. [12], [39] In the present study, we had planned to further discriminate patients with different prognosis using CD163, a relatively specific marker for the M2 macrophages but found that the peritumoral CD163^+^ cells was only associated with a poor OS in Kaplan-Meier survival analysis and its predictive value was not better than that of CD68. Therefore, CD163 is not a good biomarker to discriminate patients’ prognosis when used in immunohistochemistry studies. In other words, the CD163^+^ macrophages infiltration may play a limited role in the progression of HCC.

In glioma, the proportions of CD163^+^ macrophages among CD68^+^ macrophages, which would reflect the proportion of macrophages polarized to the M2 phenotype, were reported to be associated with histologic grade. [40] In the present study, the CD163/CD68 ratio showed the limited clinical significance. Furthermore, CD163 did not seem to be a marker of a subpopulation of macrophages if CD68 could be deemed as a surface of pan-macrophages because the CD163^+^ cell density was much higher than CD68^+^ cell density in tumor and peritumoral liver tissue. Immunostaining of consecutive sections and FCM analysis also showed that the number of CD163^+^ cells was much higher than that of CD68^+^ cells both in tumor and in peritumoral liver tissues. Therefore, we can conclude that CD163 could not be used as a marker of subpopulation of CD68^+^ cells either in liver cancer or in liver tissue.

Both plasma sCD163 and peritumoral infiltrated CD163^+^ cells could be the indicators of chronic hepatitis. Previous studies reported that sCD163 was elevated in acute liver failure and was a predictor of mortality for the patients with acute liver failure. [31], [32] In accordance with these reports, we found that plasma sCD163 was associated with inflammatory markers of hepatitis, such as serum ALT, γ-GT and ALP. The densities of peritumoral infiltrated CD163^+^ cells were also positively correlated with these markers. Plasma sCD163 level and peritumoral CD163 expression was not statistically significant difference between the patients with and without cirrhosis, which is consistent with another report showing that the sCD163 level was higher in patients with acute liver failure than in those with stable liver cirrhosis or healthy controls [32].

Tumor cells were also reported to express CD163. Shabo et al reported that cancer cells expressing CD163 were associated with poor prognosis in patients with breast cancer [41] and rectal cancer, [42] and the CD163^+^ cancer cells were deemed as the fusion of cancer cells and macrophages and could be more radiation resistant or more aggressive. We also studied the expression of CD163 on tumor cells, but found few CD163-expressing tumor cells in HCC.

Taken together, the present results showed that the CD163^+^ cell density is of limited significance to predict the prognosis of the HCC patients. Plasma sCD163 and peritumoral CD163^+^ cell infiltration was more likely a maker of active hepatitis rather than a marker of tumor progression. And the marker CD163 itself could not be used as an indicator for the determination of the subpopulation of M2-macrophages in liver and in liver cancer tissue.
